# Ultra‐High‐Molecular‐Weight Polyethylene Cables for Sternal Closure in a Metal‐Allergic Patient With Stanford Type A Acute Aortic Dissection: A Case Report

**DOI:** 10.1002/ccr3.72870

**Published:** 2026-06-05

**Authors:** Haruki Tanaka, Tasuku Kawaguchi, Junya Kimura, Masafumi Miyao, Yuki Takagi, Toru Mikoshiba, Hajime Ichimura, Taishi Fujii, Noburo Ohashi, Yuko Wada, Tatsuichiro Seto

**Affiliations:** ^1^ Division of Cardiovascular Surgery, Department of Surgery Shinshu University School of Medicine Matsumoto Japan

**Keywords:** aortic dissection, metal allergy, non‐metallic implant, sternal closure, ultra‐high molecular weight polyethylene cables

## Abstract

Ultra‐high molecular weight polyethylene cables may provide a useful and effective non‐metallic alternative for sternal closure in patients with suspected or confirmed metal hypersensitivity, particularly in emergency cardiac surgery where preoperative allergy testing is not feasible.

## 1 Introduction

Median sternotomy is often performed in cardiovascular surgery. Stainless‐steel wires are commonly used for sternal closure because of their affordability and mechanical reliability [1]. However, alternative closure methods must be considered in patients with metal hypersensitivity. Stainless steel contains nickel and chromium, which are common sensitizers in type IV hypersensitivity reactions [2, 3, 4]. Although titanium is generally biocompatible and is often utilized in patients with metal allergy [5], hypersensitivity reactions to titanium have been reported, particularly in dental and orthopedic settings [6, 7, 8].

Here, we describe a case in which ultra‐high‐molecular‐weight polyethylene (UHMW‐PE) cables were used for sternal closure in a patient with a history of metal allergy. The patient provided written informed consent for publication.

## 2 Case History/Examination

An 81‐year‐old Japanese woman who had a history of hypertension and metal hypersensitivity presented with acute nocturnal chest pain. Ten years ago, she had experienced a generalized pruritic eruption attributed to dental prostheses. Patch testing based on International Contact Dermatitis Research Group criteria revealed positive reactions to tin and zinc, whereas nickel, chromium, and iron showed negative results. The removal of the dental prostheses completely resolved all her symptoms. She had no history of diabetes mellitus or chronic obstructive pulmonary disease and no prior cardiac or thoracic surgery. Her height was 148 cm and her weight was 47 kg, corresponding to a body surface area of 1.39 m^2^.

## 3 Investigations and Treatment

**FIGURE 1:**
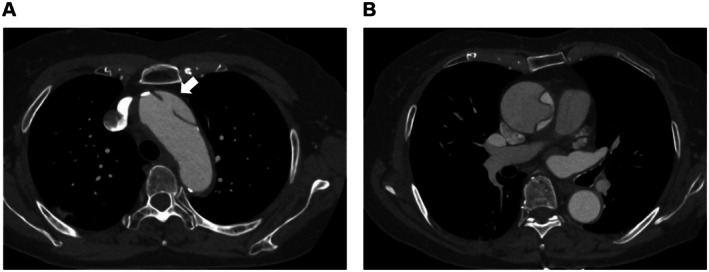
Preoperative contrast‐enhanced computed tomography. (A) The primary entry tear is located at the aortic arch (white arrow). (B) A patent false lumen is found in the ascending aorta, whereas the descending aorta remains intact.

Contrast‐enhanced computed tomography (CT) at a referring hospital revealed Stanford type A acute aortic dissection with an entry tear at the aortic arch (Figure 1). Subsequently, she was transferred to our institution for emergency surgery.

Upon admission, her blood pressure was 79/44 mmHg under antihypertensive therapy. Neurological examination was not remarkable. Transthoracic echocardiography demonstrated preserved left ventricular systolic function and a small amount of pericardial effusion without significant valvular dysfunction. CT confirmed a patent false lumen extending from the ascending aorta into the aortic arch. Thus, emergency surgical treatment was indicated.

Surgery was performed via median sternotomy. Cardiopulmonary bypass was established through the right axillary and left femoral arteries, with two‐stage venous drainage from the right atrium. At a rectal temperature of 25°C, circulatory arrest was induced, followed by selective cerebral perfusion via the left subclavian and left common carotid arteries. The entry site was identified within the aorta at the brachiocephalic artery level. A four‐branched prosthetic graft (J‐graft SHIELD NEO 24 mm, Japan Lifeline Co. Ltd., Tokyo, Japan) was implanted. Distal anastomosis was performed between the left subclavian and left common carotid arteries. Systemic circulation was resumed through a side branch of the graft, and proximal anastomosis was then performed. After the proximal anastomosis, the left common carotid and brachiocephalic arteries were anastomosed to the respective branches of the graft using 5‐0 polypropylene sutures.

**FIGURE 2:**
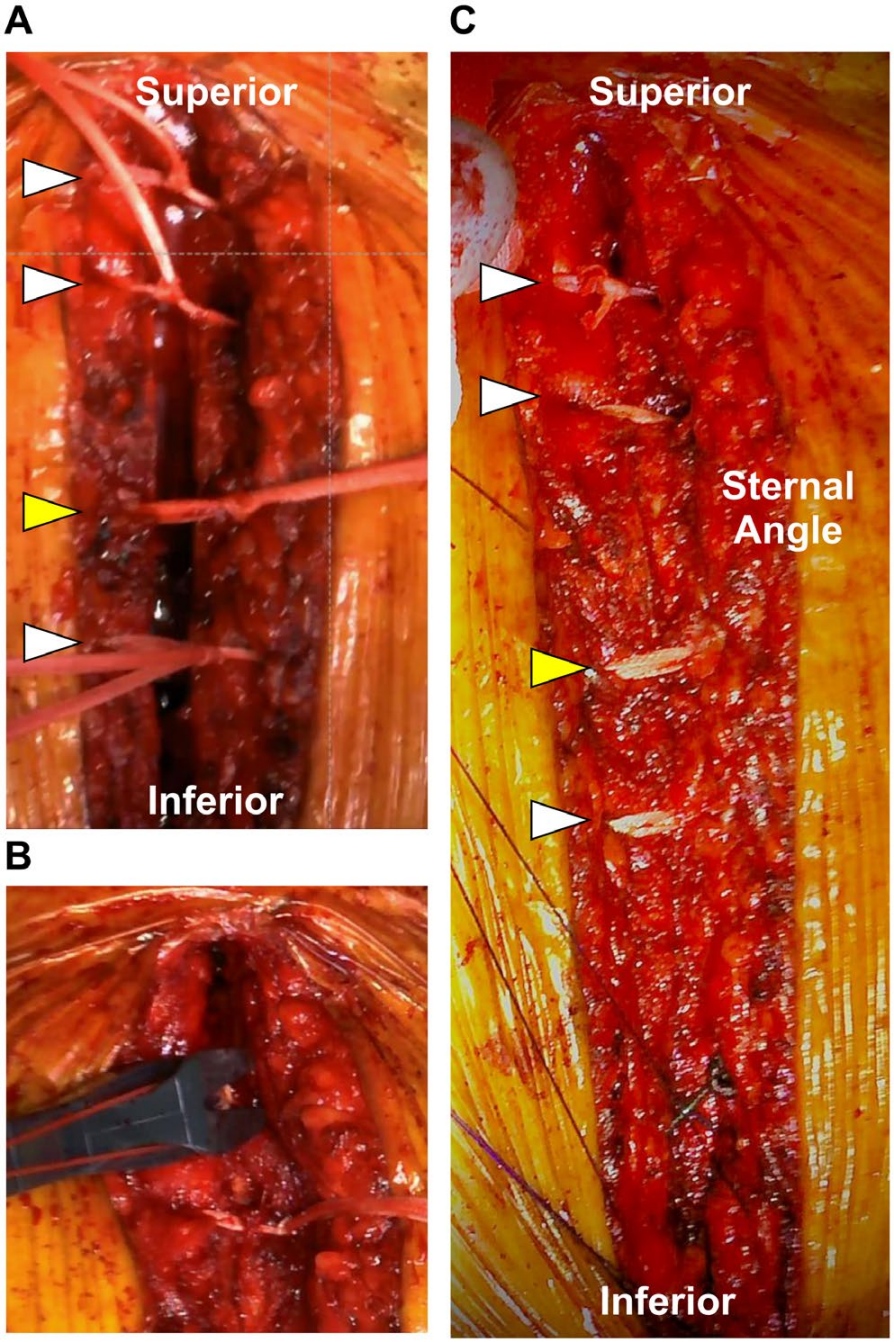
Sternal closure using ultra‐high‐molecular‐weight polyethylene (UHMW‐PE) cables. White and yellow arrowheads indicate the 3‐mm and 5‐mm UHMW‐PE cables, respectively. (A) Placement of UHMW‐PE cables around the sternum. (B) Tightening of cables using a tensioning device. (C) Final appearance following closure.

For sternal closure, two 3‐mm UHMW‐PE cables (Nespron; Alfresa Pharma Corporation, Osaka, Japan) were placed through the manubrium (Figure 2). Another 5‐mm and 3‐mm cables were placed at the second and fourth intercostal spaces, respectively. A No. 5 braided polyester suture (Ethibond, Ethicon Inc., Somerville, NJ, USA) was utilized at the fifth intercostal level. Initially, the UHMW‐PE cables were secured using a double‐loop sliding knot, followed by a standard manual knot. Each cable was then tightened using a dedicated tensioning device to a target of approximately 15 kgf; finally, it was manually knotted to ensure secure fixation. The sternum was stable upon palpation. The Nespron system is officially approved for sternal closure in Japan, indicating its suitability for this application.

## 4 Outcome and Follow‐Up

**FIGURE 3:**
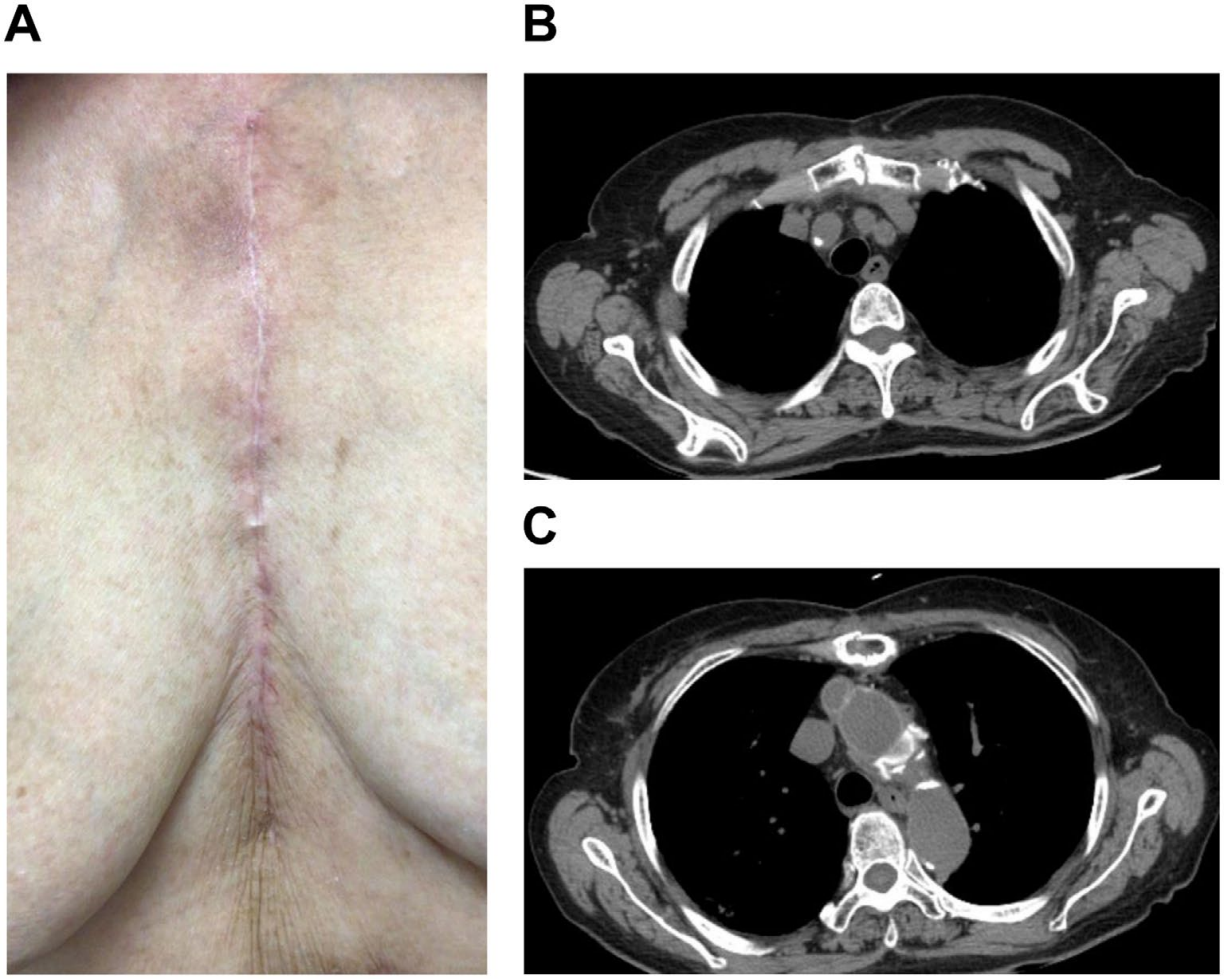
Postoperative course. (A) Skin findings 3 months postoperatively: No abnormalities were observed. (B) Computed tomography 6 months postoperatively showing no displacement of the manubrium. (C) No displacement of the sternal body.

Postoperative recovery was uneventful. The patient was extubated on postoperative Day 1 and transferred from the intensive care unit on day 2. A contrast‐enhanced CT on postoperative day 8 did not reveal abnormalities at the anastomotic or sternal sites. She was discharged on day 21. At the 3‐ and 6‐month follow‐up, no signs of wound complications or sternal dehiscence on CT were observed (Figure 3).

## 5 Discussion

Stainless‐steel wires are still the gold standard for sternal closure following median sternotomy owing to their low cost, ease of use, and reliable mechanical performance [1]. However, nickel and chromium, stainless steel components, are well‐recognized sensitizers in type IV hypersensitivity reactions [2, 3, 9]. Despite the rarity of true allergic reactions to surgical stainless steel (type 316 L), a study reported wound pain, granuloma formation, and erythema attributable to metal allergy in the context of sternal closure [5].

In this patient, patch testing performed a decade earlier revealed sensitization to tin and zinc, whereas nickel, chromium, and iron showed negative results. However, the possibility of false‐negative results cannot be completely excluded, and sensitization profiles may change over time [3, 4]. Given the emergency setting and inability to repeat patch testing, a nonmetallic closure method was employed to minimize any potential risk of hypersensitivity.

Titanium wires are often used in patients with metal allergy because of their high corrosion resistance and generally favorable biocompatibility [5]. However, rare cases of titanium hypersensitivity have been reported, particularly in dentistry and orthopedics [6, 7, 8]. Despite the lack of confirmed cases of allergic reactions to titanium sternal closure devices, such as wires and plates, the possibility, albeit low, could not be excluded in the presented patient, who had never undergone titanium‐specific patch testing.

Polyester‐based sutures, such as Ethibond, are occasionally used for sternal closure in patients with metal allergy. However, their lower rigidity has raised issues about the risk of sternal dehiscence [9].

Conversely, UHMW‐PE cables, such as the Nespron system, combine high tensile strength with flexibility and a flat profile, which distributes pressure more evenly across the sternum. Khalpey et al. reported that compared with conventional stainless‐steel wires, UHMW‐PE cables were associated with significantly lower rates of sternal dehiscence and infections and minimal postoperative pain [10]. These findings support the clinical efficacy of UHMW‐PE materials. Notably, the Nespron cable used in the present case is wider than that described in a previous report, with available widths of 3 and 5 mm, which may further decrease sternal cutting risk by distributing pressure more broadly across the sternal surface. In this case, UHMW‐PE cables were placed in a simple transverse configuration. Subsequently, based on previous reports, including the study by Khalpey et al., we modified our technique and currently apply two UHMW‐PE cables in a figure‐of‐eight configuration in the sternal body, with one placed through the second‐to‐third intercostal space and the other through the fourth‐to‐fifth intercostal space.

In our institution, UHMW‐PE cables are not routinely used in all patients; they are used adjunctively in patients with fragile sternal bone, such as those with osteoporosis, and are primarily used in patients with suspected or confirmed metal hypersensitivity. To date, we have performed sternal closure using only UHMW‐PE cables (Nespron) in two patients with metal hypersensitivity, including the present case, and no cases of sternal dehiscence or surgical site infection have been observed.

Furthermore, given their nonmetallic nature and favorable handling characteristics, UHMW‐PE cables have been employed in other thoracic procedures, such as sternoclavicular joint dislocation repair, achieving satisfactory outcomes [11]. Importantly, the Nespron cable is an approved device for sternal closure in Japan; thus, its application in the present case was not off‐label. UHMW‐PE cables represent a promising substitute for sternal closure, particularly in patients with suspected or confirmed metal hypersensitivity and even in the broader population, particularly those with fragile bones, such as osteoporosis.

This report describes our experience of a case in which UHMW‐PE cables were successfully used for sternal closure in a patient with Stanford type A acute aortic dissection and history of metal allergy. The postoperative course was uneventful, and no evidence of wound complications or sternal instability was observed during follow‐up. Thus, UHMW‐PE cables appear to be a viable substitute for sternal closure in patients with metal hypersensitivity, particularly in emergency settings where preoperative allergy testing is not feasible. However, further studies are warranted to assess their long‐term safety and mechanical durability.

## Author Contributions

**Haruki Tanaka:** conceptualization, investigation, writing – original draft. **Tasuku Kawaguchi:** data curation. **Junya Kimura:** data curation. **Masafumi Miyao:** investigation. **Yuki Takagi:** investigation. **Toru Mikoshiba:** investigation. **Taishi Fujii:** investigation. **Hajime Ichimura:** supervision, writing – review and editing. **Yuko Wada:** data curation. **Tatsuichiro Seto:** supervision, writing – review and editing. **Noburo Ohashi:** validation.

## Funding

The authors have nothing to report.

## Ethics Statement

This case report did not require approval from an ethics committee because it describes a single clinical case without experimental intervention. The study was conducted in accordance with the Declaration of Helsinki, and written informed consent was obtained from the patient.

## Consent

Written informed consent was obtained from the patient for publication of this case report and accompanying images.

## Conflicts of Interest

The authors declare no conflicts of interest.

## Data Availability

Data sharing is not applicable to this article as no datasets were generated or analyzed during the current study.

## References

[1] A. Nenna , F. Nappi , J. Dougal , et al., “Sternal Wound Closure in the Current Era: The Need of a Tailored Approach,” General Thoracic and Cardiovascular Surgery 67 (2019): 907–916, 10.1007/s11748-019-01204-5. 31531834

[2] M. Saito , R. Arakaki , A. Yamada , T. Tsunematsu , Y. Kudo , and N. Ishimaru , “Molecular Mechanisms of Nickel Allergy,” International Journal of Molecular Sciences 17 (2016): 202, 10.3390/ijms17020202. 26848658 PMC4783936

[3] M.‐E. Tu and Y.‐H. Wu , “Multiple Allergies to Metal Alloys,” Dermatologica Sinica 29 (2011): 41–43, 10.1016/j.dsi.2011.05.010.

[4] M. Zemelka‐Wiacek , “Metal Allergy: State‐Of‐The‐Art Mechanisms, Biomarkers, Hypersensitivity to Implants,” Journal of Clinical Medicine 11 (2022): 6971, 10.3390/jcm11236971. 36498546 PMC9739320

[5] M. Eraqi , A. H. Diab , K. Matschke , and K. Alexiou , “Confirmation of Safety of Titanium Wire in Sternotomy Closure, a Randomized Prospective Study,” Thoracic and Cardiovascular Surgeon 72 (2024): 70–76, 10.1055/s-0043-1764315. 36918153 PMC10786665

[6] S. W. Fage , J. Muris , S. S. Jakobsen , and J. P. Thyssen , “Titanium: A Review on Exposure, Release, Penetration, Allergy, Epidemiology, and Clinical Reactivity,” Contact Dermatitis 74 (2016): 323–345, 10.1111/cod.12565. 27027398

[7] P. P. Poli , F. V. de Miranda , T. O. B. Polo , et al., “Titanium Allergy Caused by Dental Implants: A Systematic Literature Review and Case Report,” Materials 14 (2021): 5239, 10.3390/ma14185239. 34576463 PMC8465040

[8] J. Voves , O. Merka , K. Cabanova , J. Janosek , and G. Bajor , “Type IV Titanium Hypersensitivity: Rare, or Rarely Detected?,” Acta Chirurgiae Orthopaedicae et Traumatologiae Cechoslovaca 90 (2023): 85–91. 37155996

[9] A. R. Casha , L. Yang , P. H. Kay , M. Saleh , and G. J. Cooper , “A Biomechanical Study of Median Sternotomy Closure Techniques,” European Journal of Cardio‐Thoracic Surgery 15 (1999): 365–369, 10.1016/s1010-7940(99)00014-7. 10333037

[10] Z. Khalpey , U. A. Kumar , U. Aslam , et al., “Improving Sternal Closure Outcomes in Cardiac Surgery: Polyethylene Suture Tapes vs. Steel Wires,” Journal of Clinical Medicine 14 (2025): 277, 10.3390/jcm14010277. 39797360 PMC11720976

[11] H. Takahashi , S. Takeda , R. Shibata , et al., “Posterior Sternoclavicular Joint Dislocation With Thoracic Costovertebral Joints Fracture‐Dislocations: A Case Report,” Trauma Case Reports 43 (2023): 100766, 10.1016/j.tcr.2023.100766. 36718405 PMC9883233

